# A new monoclinic polymorph of 3-diethyl­amino-4-(4-meth­oxy­phen­yl)-1,1-dioxo-4*H*-1λ^6^,2-thia­zete-4-carbonitrile

**DOI:** 10.1107/S1600536810027558

**Published:** 2010-07-17

**Authors:** Ahmed M. Orlando, Leonardo Lo Presti, Raffaella Soave

**Affiliations:** a Dipartimento di Chimica Fisica ed Elettrochimica, Universitá degli Studi di Milano, Via Golgi 19, 20133 Milano, Italy; b Consiglio Nazionale delle Ricerche, Istituto di Scienze e Tecnologie Molecolari, Via Golgi 19, 20133 Milano, Italy

## Abstract

A new monoclinic form of the title compound, C_14_H_17_N_3_O_3_S, has been found upon slow crystallization from water. Another monoclinic form of the compound was obtained previously from a mixture of dichloro­methane and diethyl ether [Clerici *et al.* (2002 ▶). *Tetra­hedron*, **58**, 5173–5178]. Both phases crystallize in space group *P*2_1_/*n* with one mol­ecule in the asymmetric unit. The formally single exocyclic C—N bond that connects the –NEt_2_ unit with the thia­zete ring is considerably shorter than the adjacent, formally double, endocyclic C=N bond. This is likely to be due to the extended conjugated system between the electron-donor diethyl­ammine fragment and the electron-withdrawing sulfonyl group. In the newly discovered polymorph, the meth­oxy group is rotated by almost 180° around the phen­yl–OCH_3_ bond, resulting in a different mol­ecular conformation.

## Related literature

For the synthesis of the title compound and the crystal structure of the other polymorph, see: Clerici *et al.* (2002 ▶). For a related structure, see: Clerici *et al.* (1996 ▶). For the biological activity of β-sultam derivatives, see: Barwick *et al.* (2008 ▶) and references therein.
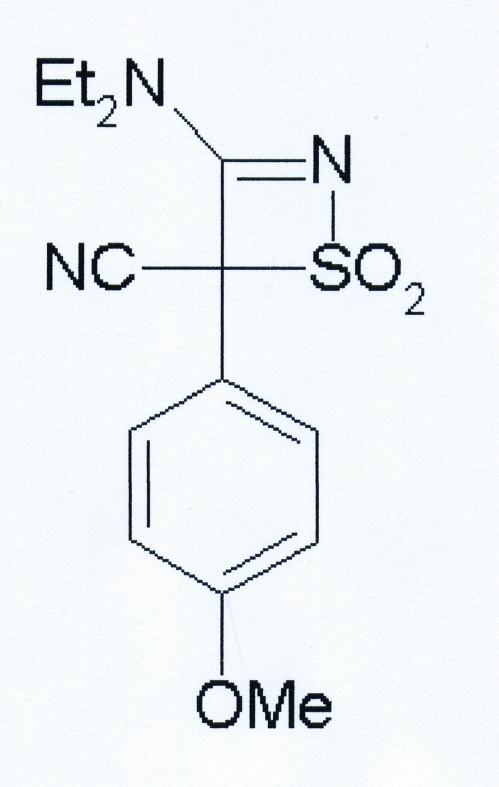


## Experimental

### 

#### Crystal data

C_14_H_17_N_3_O_3_S
*M* *_r_* = 307.37
Monoclinic, 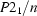

*a* = 8.3853 (17) Å
*b* = 17.554 (4) Å
*c* = 10.458 (2) Å
β = 95.07 (3)°
*V* = 1533.4 (5) Å^3^
*Z* = 4
Mo *K*α radiation
μ = 0.22 mm^−1^
*T* = 293 K
0.18 × 0.16 × 0.16 mm

#### Data collection

Bruker APEX CCD area-detector diffractometer
Absorption correction: multi-scan (*SADABS*; Bruker, 2007 ▶) *T*_min_ = 0.855, *T*_max_ = 0.947
16661 measured reflections
2814 independent reflections
1949 reflections with *I* > 2σ(*I*)
*R*_int_ = 0.043

#### Refinement

*R*[*F* ^2^ > 2σ(*F* ^2^)] = 0.037
*wR*(*F* ^2^) = 0.102
*S* = 1.01
2814 reflections
258 parameters
All H-atom parameters refined
Δρ_max_ = 0.16 e Å^−3^
Δρ_min_ = −0.31 e Å^−3^

### 

Data collection: *SMART* (Bruker, 2005 ▶); cell refinement: *SAINT* (Bruker, 2005 ▶); data reduction: *SAINT*; program(s) used to solve structure: *SHELXS97* (Sheldrick, 2008 ▶); program(s) used to refine structure: *SHELXL97* (Sheldrick, 2008 ▶); molecular graphics: *DIAMOND* (Brandenburg, 2010 ▶); software used to prepare material for publication: *SHELXL97*.

## Supplementary Material

Crystal structure: contains datablocks global, I. DOI: 10.1107/S1600536810027558/nk2045sup1.cif

Structure factors: contains datablocks I. DOI: 10.1107/S1600536810027558/nk2045Isup2.hkl

Additional supplementary materials:  crystallographic information; 3D view; checkCIF report

## Figures and Tables

**Fig. 1.**
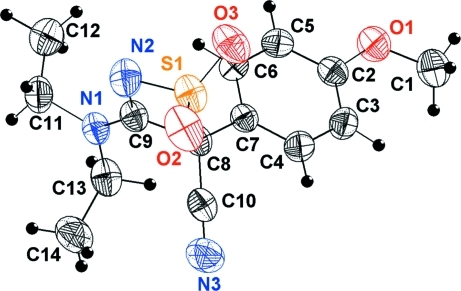
Molecular structure of (I), with the non-H atom numbering scheme. Displacement ellipsoids are drawn at the 50% probability level.

**Fig. 2.**
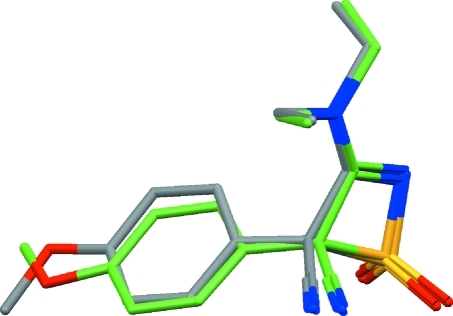
Least-squares overlay scheme of the asymmetric units of (I) within phase B (this work, carbon backbone in gray) and phase A (Clerici et al., 2002; carbon backbone in green). Hydrogen atoms omitted for clarity.

## supplementary crystallographic information

### Comment

The title compound, (I), a thiazete 1,1 dioxo derivative containing a four-membered heterocycle, exhibits a marked similarity with the β-sultamic functionality, which is the key component of promising antibiotic drugs (Barwick *et al.*, 2008). A new monoclinic polymorph of (I) (hereinafter, phase B: Fig. 1, Table 1) was found upon slow recrystallization from water of a little amount of the phase A, originally obtained from a CH_2_Cl_2_:Et_2_O mixture (Clerici *et al.*, 2002). Both polymorphs share the same space group, *P*2_1_/*n*, with one molecule in the asymmetric unit. On average, bond lengths and angles are very similar between the two forms, while the molecular conformations are different. The most important dissimilarity resides in the dihedral angles involving the phenyl-OCH_3_ single bond, which is rotated by ~180° in the form B with respect to form A (Fig. 2). In both crystal forms the formally single exocyclic C9–N1 bond connecting the –NEt_2_ moiety to the thiazete ring is considerably shorter (phase B: 1.307 (3) Å; phase A: 1.318 (3) Å) than the adjacent, formally double, endocyclic C9=N2 bond (phase B: 1.331 (3) Å; phase A: 1.327 (3) Å). A possible explanation resides in the existence of an extended π conjugated system between the electron-donor diethylammine fragment and the electron-withdrawing sulfonyl group. This conjecture is supported by the values of the C–N1–C angles, which in both phases range from ~118° to ~122° and are compatible with a formally *sp*^2^ tertiary nitrogen atom. Very similar bond distances within the thiazete group have been reported by Clerici *et al.* (1996) for a chemically related derivative of (I). On geometrical grounds, no relevant intermolecular hydrogen bonds have been found in both phases.

### Experimental

The compound (I) was synthesized using the procedure reported by Clerici *et al.* (2002). Part of the material obtained from dichloromethane and diethyl ether (phase A) was dissolved in distilled water and crystallized by slow solvent evaporation at room temperature. After roughly 7 days, very small colorless crystals with the same habit (prism) as the most common phase A appeared. Only the X-ray analysis revealed that in fact a new polymorph (phase B) was obtained.

### Refinement

All hydrogen atoms have been located by difference Fourier. Data collection: *SMART* (Bruker, 2005); cell refinement: *SAINT* (Bruker, 2005); data reduction: *SAINT* (Bruker, 2005); absorption correction: *SADABS* (Bruker, 2007); program used to solve structure: *SHELXS97* (Sheldrick, 2008); program used to refine structure: *SHELXL97* (Sheldrick, 2008); molecular graphic: *DIAMOND* (Brandenburg, 2010); overlay scheme: Mercury CSD 2.3

### Crystal data

**Table:**

C_14_H_17_N_3_O_3_S	*F*(000) = 648
*M**_r_* = 307.37	*D*_x_ = 1.331 Mg m^−^^3^
Monoclinic, *P*2_1_/*n*	Mo *K*α radiation, λ = 0.71073 Å
Hall symbol: -P 2yn	Cell parameters from 2885 reflections
*a* = 8.3853 (17) Å	θ = 2.3–21.6°
*b* = 17.554 (4) Å	µ = 0.22 mm^−^^1^
*c* = 10.458 (2) Å	*T* = 293 K
β = 95.07 (3)°	Prism, colourless
*V* = 1533.4 (5) Å^3^	0.18 × 0.16 × 0.16 mm
*Z* = 4	

### Data collection

**Table:**

Bruker APEX CCD area-detector diffractometer	2814 independent reflections
Radiation source: fine-focus sealed tube	1949 reflections with *I* > 2σ(*I*)
graphite	*R*_int_ = 0.043
ω scans	θ_max_ = 25.4°, θ_min_ = 2.3°
Absorption correction: multi-scan (*SADABS*; Bruker, 2007)	*h* = −10→10
*T*_min_ = 0.855, *T*_max_ = 0.947	*k* = −21→21
16661 measured reflections	*l* = −12→12

### Refinement

**Table:**

Refinement on *F*^2^	Primary atom site location: structure-invariant direct methods
Least-squares matrix: full	Secondary atom site location: structure-invariant direct methods
*R*[*F*^2^ > 2σ(*F*^2^)] = 0.037	Hydrogen site location: difference Fourier map
*wR*(*F*^2^) = 0.102	All H-atom parameters refined
*S* = 1.01	*w* = 1/[σ^2^(*F*_o_^2^) + (0.0446*P*)^2^ + 0.4078*P*] where *P* = (*F*_o_^2^ + 2*F*_c_^2^)/3
2814 reflections	(Δ/σ)_max_ < 0.001
258 parameters	Δρ_max_ = 0.16 e Å^−^^3^
0 restraints	Δρ_min_ = −0.31 e Å^−^^3^

### Special details

**Table:**

Geometry. All e.s.d.'s (except the e.s.d. in the dihedral angle between two l.s. planes) are estimated using the full covariance matrix. The cell e.s.d.'s are taken into account individually in the estimation of e.s.d.'s in distances, angles and torsion angles; correlations between e.s.d.'s in cell parameters are only used when they are defined by crystal symmetry. An approximate (isotropic) treatment of cell e.s.d.'s is used for estimating e.s.d.'s involving l.s. planes.
Refinement. Refinement of *F*^2^ against ALL reflections. The weighted *R*-factor *wR* and goodness of fit *S* are based on *F*^2^, conventional *R*-factors *R* are based on *F*, with *F* set to zero for negative *F*^2^. The threshold expression of *F*^2^ > σ(*F*^2^) is used only for calculating *R*-factors(gt) *etc*. and is not relevant to the choice of reflections for refinement. *R*-factors based on *F*^2^ are statistically about twice as large as those based on *F*, and *R*- factors based on ALL data will be even larger.

### Fractional atomic coordinates and isotropic or equivalent isotropic displacement parameters (Å^2^)

**Table:**

	*x*	*y*	*z*	*U*_iso_*/*U*_eq_	
S1	0.46791 (6)	0.19850 (4)	0.39836 (6)	0.0569 (2)	
O1	0.1824 (2)	0.00687 (9)	−0.10877 (14)	0.0636 (5)	
O2	0.57694 (19)	0.19314 (11)	0.51054 (16)	0.0758 (5)	
O3	0.53410 (19)	0.20903 (10)	0.27881 (16)	0.0724 (5)	
N1	0.0608 (2)	0.20205 (10)	0.44220 (16)	0.0472 (4)	
N2	0.3156 (2)	0.25469 (11)	0.41859 (19)	0.0611 (5)	
N3	0.3384 (3)	0.02616 (14)	0.5764 (2)	0.0739 (6)	
C1	0.2489 (4)	−0.06433 (17)	−0.1428 (3)	0.0698 (8)	
C2	0.2106 (2)	0.03021 (12)	0.01541 (19)	0.0458 (5)	
C3	0.2867 (3)	−0.01266 (14)	0.1123 (2)	0.0521 (6)	
C4	0.3129 (3)	0.01743 (13)	0.2346 (2)	0.0491 (5)	
C5	0.1573 (3)	0.10253 (13)	0.0415 (2)	0.0497 (5)	
C6	0.1842 (3)	0.13239 (13)	0.1623 (2)	0.0474 (5)	
C7	0.2636 (2)	0.09019 (11)	0.26096 (18)	0.0395 (5)	
C8	0.3043 (2)	0.12520 (12)	0.39177 (19)	0.0425 (5)	
C9	0.2122 (2)	0.19738 (12)	0.42216 (19)	0.0448 (5)	
C10	0.3242 (2)	0.06955 (14)	0.4954 (2)	0.0496 (5)	
C11	−0.0148 (3)	0.27758 (15)	0.4525 (3)	0.0599 (7)	
C12	−0.0724 (4)	0.3089 (2)	0.3236 (3)	0.0769 (8)	
C13	−0.0377 (3)	0.13436 (15)	0.4614 (2)	0.0553 (6)	
C14	−0.0494 (4)	0.1173 (2)	0.6015 (3)	0.0780 (9)	
H1A	0.226 (3)	−0.0650 (17)	−0.234 (3)	0.105 (10)*	
H1B	0.201 (3)	−0.1077 (16)	−0.096 (3)	0.085 (9)*	
H1C	0.364 (3)	−0.0633 (15)	−0.122 (2)	0.081 (9)*	
H3	0.317 (3)	−0.0621 (14)	0.100 (2)	0.066 (7)*	
H4	0.367 (2)	−0.0118 (12)	0.299 (2)	0.055 (6)*	
H5	0.100 (2)	0.1321 (12)	−0.027 (2)	0.055 (6)*	
H6	0.153 (3)	0.1815 (13)	0.176 (2)	0.056 (7)*	
H11A	−0.101 (3)	0.2705 (13)	0.502 (2)	0.068 (7)*	
H11B	0.064 (3)	0.3094 (15)	0.495 (2)	0.078 (9)*	
H12A	−0.122 (3)	0.3602 (18)	0.334 (3)	0.095 (9)*	
H12B	−0.152 (4)	0.2716 (19)	0.279 (3)	0.114 (12)*	
H12C	0.016 (4)	0.3143 (15)	0.271 (3)	0.088 (9)*	
H13A	−0.139 (3)	0.1458 (12)	0.4177 (19)	0.053 (6)*	
H13B	0.008 (2)	0.0906 (13)	0.4177 (19)	0.052 (6)*	
H14A	−0.113 (4)	0.073 (2)	0.613 (3)	0.126 (12)*	
H14B	−0.094 (3)	0.1583 (18)	0.642 (3)	0.095 (10)*	
H14C	0.062 (4)	0.1065 (18)	0.653 (3)	0.116 (12)*	

### Atomic displacement parameters (Å^2^)

**Table:**

	*U*^11^	*U*^22^	*U*^33^	*U*^12^	*U*^13^	*U*^23^
S1	0.0428 (3)	0.0691 (4)	0.0599 (4)	−0.0149 (3)	0.0108 (3)	−0.0137 (3)
O1	0.0840 (12)	0.0642 (11)	0.0414 (9)	0.0047 (9)	−0.0024 (8)	−0.0079 (8)
O2	0.0492 (9)	0.1081 (15)	0.0690 (11)	−0.0171 (9)	−0.0014 (8)	−0.0233 (10)
O3	0.0624 (10)	0.0883 (13)	0.0705 (11)	−0.0220 (9)	0.0270 (9)	−0.0068 (10)
N1	0.0434 (10)	0.0506 (11)	0.0489 (10)	−0.0011 (8)	0.0116 (8)	−0.0073 (8)
N2	0.0559 (12)	0.0537 (12)	0.0755 (14)	−0.0126 (9)	0.0162 (10)	−0.0154 (10)
N3	0.0798 (15)	0.0884 (17)	0.0534 (13)	0.0113 (13)	0.0047 (11)	0.0129 (12)
C1	0.092 (2)	0.0672 (19)	0.0505 (17)	−0.0020 (17)	0.0059 (15)	−0.0149 (14)
C2	0.0466 (12)	0.0514 (13)	0.0392 (12)	−0.0049 (10)	0.0033 (9)	−0.0013 (10)
C3	0.0619 (14)	0.0448 (14)	0.0492 (13)	0.0077 (11)	0.0032 (10)	−0.0051 (11)
C4	0.0510 (13)	0.0524 (14)	0.0428 (13)	0.0089 (11)	−0.0011 (10)	0.0024 (11)
C5	0.0563 (13)	0.0506 (14)	0.0417 (12)	0.0046 (11)	0.0010 (10)	0.0066 (11)
C6	0.0517 (13)	0.0415 (13)	0.0495 (14)	0.0046 (10)	0.0072 (10)	0.0021 (11)
C7	0.0355 (10)	0.0430 (12)	0.0405 (11)	−0.0034 (9)	0.0066 (8)	−0.0013 (9)
C8	0.0371 (11)	0.0492 (13)	0.0415 (12)	−0.0022 (9)	0.0054 (9)	−0.0042 (10)
C9	0.0437 (11)	0.0491 (13)	0.0422 (12)	−0.0050 (10)	0.0064 (9)	−0.0074 (10)
C10	0.0447 (12)	0.0619 (15)	0.0421 (13)	0.0007 (11)	0.0040 (10)	−0.0062 (12)
C11	0.0602 (16)	0.0581 (16)	0.0635 (17)	0.0092 (13)	0.0169 (13)	−0.0099 (13)
C12	0.083 (2)	0.073 (2)	0.077 (2)	0.0205 (18)	0.0162 (17)	0.0058 (17)
C13	0.0408 (13)	0.0619 (16)	0.0640 (16)	−0.0094 (11)	0.0095 (11)	−0.0148 (13)
C14	0.085 (2)	0.078 (2)	0.077 (2)	−0.0188 (19)	0.0353 (18)	−0.0019 (17)

### Geometric parameters (Å, °)

**Table:**

S1—O3	1.4241 (17)	C5—C6	1.369 (3)
S1—O2	1.4252 (18)	C5—H5	0.98 (2)
S1—N2	1.642 (2)	C6—C7	1.391 (3)
S1—C8	1.878 (2)	C6—H6	0.92 (2)
O1—C2	1.362 (2)	C7—C8	1.511 (3)
O1—C1	1.426 (3)	C8—C10	1.457 (3)
N1—C9	1.307 (3)	C8—C9	1.532 (3)
N1—C13	1.471 (3)	C11—C12	1.496 (4)
N1—C11	1.478 (3)	C11—H11A	0.94 (2)
N2—C9	1.331 (3)	C11—H11B	0.95 (3)
N3—C10	1.138 (3)	C12—H12A	1.00 (3)
C1—H1A	0.96 (3)	C12—H12B	1.02 (3)
C1—H1B	1.01 (3)	C12—H12C	0.97 (3)
C1—H1C	0.97 (3)	C13—C14	1.508 (4)
C2—C3	1.373 (3)	C13—H13A	0.95 (2)
C2—C5	1.381 (3)	C13—H13B	0.99 (2)
C3—C4	1.384 (3)	C14—H14A	0.95 (4)
C3—H3	0.92 (2)	C14—H14B	0.93 (3)
C4—C7	1.377 (3)	C14—H14C	1.06 (3)
C4—H4	0.94 (2)		
			
O3—S1—O2	117.38 (11)	C10—C8—C7	113.72 (18)
O3—S1—N2	113.77 (11)	C10—C8—C9	115.25 (17)
O2—S1—N2	112.54 (11)	C7—C8—C9	116.52 (17)
O3—S1—C8	113.38 (10)	C10—C8—S1	113.39 (14)
O2—S1—C8	113.47 (10)	C7—C8—S1	114.70 (13)
N2—S1—C8	80.92 (9)	C9—C8—S1	78.81 (12)
C2—O1—C1	117.5 (2)	N1—C9—N2	127.0 (2)
C9—N1—C13	122.43 (19)	N1—C9—C8	126.87 (18)
C9—N1—C11	119.8 (2)	N2—C9—C8	106.11 (17)
C13—N1—C11	117.72 (19)	N3—C10—C8	179.4 (2)
C9—N2—S1	93.77 (15)	N1—C11—C12	111.7 (2)
O1—C1—H1A	102.2 (18)	N1—C11—H11A	106.2 (15)
O1—C1—H1B	111.1 (15)	C12—C11—H11A	110.1 (15)
H1A—C1—H1B	115 (2)	N1—C11—H11B	106.1 (16)
O1—C1—H1C	109.3 (16)	C12—C11—H11B	111.2 (16)
H1A—C1—H1C	109 (2)	H11A—C11—H11B	111 (2)
H1B—C1—H1C	110 (2)	C11—C12—H12A	109.8 (16)
O1—C2—C3	124.6 (2)	C11—C12—H12B	108.9 (18)
O1—C2—C5	115.63 (19)	H12A—C12—H12B	111 (2)
C3—C2—C5	119.7 (2)	C11—C12—H12C	110.2 (17)
C2—C3—C4	119.9 (2)	H12A—C12—H12C	109 (2)
C2—C3—H3	122.2 (15)	H12B—C12—H12C	108 (2)
C4—C3—H3	117.9 (15)	N1—C13—C14	112.2 (2)
C7—C4—C3	120.9 (2)	N1—C13—H13A	104.7 (13)
C7—C4—H4	120.0 (13)	C14—C13—H13A	112.1 (13)
C3—C4—H4	119.1 (13)	N1—C13—H13B	108.5 (12)
C6—C5—C2	120.3 (2)	C14—C13—H13B	110.8 (12)
C6—C5—H5	120.4 (12)	H13A—C13—H13B	108.2 (17)
C2—C5—H5	119.3 (12)	C13—C14—H14A	111 (2)
C5—C6—C7	120.7 (2)	C13—C14—H14B	110.7 (19)
C5—C6—H6	118.4 (14)	H14A—C14—H14B	109 (3)
C7—C6—H6	120.8 (14)	C13—C14—H14C	113.6 (17)
C4—C7—C6	118.55 (19)	H14A—C14—H14C	106 (3)
C4—C7—C8	120.69 (18)	H14B—C14—H14C	106 (3)
C6—C7—C8	120.64 (19)		
			
O3—S1—N2—C9	116.10 (15)	N2—S1—C8—C10	−116.78 (16)
O2—S1—N2—C9	−107.28 (15)	O3—S1—C8—C7	−1.80 (19)
C8—S1—N2—C9	4.46 (13)	O2—S1—C8—C7	−139.02 (15)
C1—O1—C2—C3	−5.7 (3)	N2—S1—C8—C7	110.27 (16)
C1—O1—C2—C5	174.0 (2)	O3—S1—C8—C9	−116.00 (13)
O1—C2—C3—C4	178.3 (2)	O2—S1—C8—C9	106.78 (13)
C5—C2—C3—C4	−1.5 (3)	N2—S1—C8—C9	−3.94 (12)
C2—C3—C4—C7	−0.1 (3)	C13—N1—C9—N2	−171.6 (2)
O1—C2—C5—C6	−177.87 (19)	C11—N1—C9—N2	5.4 (3)
C3—C2—C5—C6	1.9 (3)	C13—N1—C9—C8	11.5 (3)
C2—C5—C6—C7	−0.8 (3)	C11—N1—C9—C8	−171.5 (2)
C3—C4—C7—C6	1.2 (3)	S1—N2—C9—N1	176.98 (19)
C3—C4—C7—C8	−174.95 (19)	S1—N2—C9—C8	−5.62 (17)
C5—C6—C7—C4	−0.7 (3)	C10—C8—C9—N1	−66.9 (3)
C5—C6—C7—C8	175.38 (19)	C7—C8—C9—N1	70.2 (3)
C4—C7—C8—C10	−28.1 (3)	S1—C8—C9—N1	−177.6 (2)
C6—C7—C8—C10	155.89 (18)	C10—C8—C9—N2	115.7 (2)
C4—C7—C8—C9	−165.80 (18)	C7—C8—C9—N2	−107.2 (2)
C6—C7—C8—C9	18.2 (3)	S1—C8—C9—N2	5.00 (15)
C4—C7—C8—S1	104.7 (2)	C9—N1—C11—C12	85.4 (3)
C6—C7—C8—S1	−71.3 (2)	C13—N1—C11—C12	−97.4 (3)
O3—S1—C8—C10	131.15 (16)	C9—N1—C13—C14	96.4 (3)
O2—S1—C8—C10	−6.06 (19)	C11—N1—C13—C14	−80.6 (3)
